# Potential Impact of Microbial Dysbiosis and Tryptophan Metabolites in Advanced Stages of Colorectal Cancer

**DOI:** 10.3390/biomedicines14010026

**Published:** 2025-12-22

**Authors:** Anne Hulin, Aline Rifflet, Florence Castelli, Quentin Giai Gianetto, François Fenaille, Abdel Aissat, Mariette Matondo, Soraya Fellahi, Christophe Tournigand, Christophe Junot, Philippe Sansonetti, Ivo Gomperts-Boneca, Denis Mestivier, Iradj Sobhani

**Affiliations:** 1 EC2M3-EA7375 Unit, Université Paris Est-Créteil-UPEC, 94000 Créteil, France; 2 Département de Biochimie et Pharmacologie, DMU Biologie Pathologie, Assistance Publique Hôpitaux de Paris-APHP, Hôpital Henri Mondor, 94000 Créteil, France; 3 Biologie et Génétique de la Paroi Bactérienne, UMR/Inserm U1306, Institut Pasteur, 75000 Paris, France; 4 Département Médicaments et Technologies pour la Santé (DMTS), MetaboHUB, CEA, INRAE, Université Paris-Saclay, 91191 Gif-sur-Yvette, Francefrancois.fenaille@cea.fr (F.F.);; 5 Plateforme de Protéomique, CNRS UAR 2024, Institut Pasteur, 75000 Paris, France; 6 Département de Génétique et Biologie Moléculaire, DMU Biologie Pathologie, Assistance Publique Hôpitaux de Paris-APHP, Hôpital Henri Mondor, 94000 Créteil, France; 7 Service d’Oncologie Médicale, Assistance Publique Hôpitaux de Paris-APHP, Hôpital Henri Mondor, 94000 Créteil, France; 8 Pathologies Moléculaires Microbiennes, UMR/Inserm U786, Institut Pasteur, 75000 Paris, France; 9 Service de Gastroentérologie, Assistance Publique Hôpitaux de Paris-APHP, Hôpital Henri Mondor, 94000 Créteil, France

**Keywords:** metabolite, colon, cancer, microbiota

## Abstract

**Background/Objectives**: We conducted an untargeted metabolomic study in serum, urine, and fecal water in colorectal cancer (CRC) patients compared to healthy controls. The aim was to define the interactions between metabolites and microbiota. **Methods**: Effluents were collected before colonoscopy. Metabolites were analyzed using LC-HRMS. Bioinformatics analyses included Limma test, along with spectral house and public databases for annotations. Whole-genome shotgun sequencing was performed on fecal samples. Species–metabolite interactions were calculated using Spearman correlation. Interleukins and inflammatory proteins were measured. **Results**: Fifty-three patients (11 stage I, 10 stage II, 10 stage III, and 22 stage IV) and twenty controls were included. Derivatives of deoxycholic acid, cholic acid, and fatty acids were lower in serum, while urinary bile acids were higher in stage IV CRC patients (versus controls). Metabolites related to tryptophan and glutamate were found significantly altered in stage IV: upregulation of kynurenine and downregulation of indole pathways. This was linked to increased inflammatory protein and microbial metabolites and to the imbalance between virulent pro-inflammatory bacteria (*Escherichia* and *Desulfovibrio*) and symbiotic (*Ruminococcus* and *Bifidobacterium*) bacteria. **Conclusions**: *E. coli*-related tryptophan catabolism shift is shown through stage IV CRC as compared to controls. As a consequence, tryptophan/kynurenine metabolite may become a promising marker for detecting the failure to immune response during therapy.

## 1. Introduction

Colorectal cancer (CRC) is among the leading causes of cancer-related deaths worldwide [1]. While CRC is predominantly diagnosed in individuals over the age of 60, its incidence is constantly rising among younger adults globally [2]. This trend has led to the hypothesis that environmental changes may be contributing to the increasing prevalence of CRC. A more comprehensive understanding of host–environment interactions linked with colon carcinogenesis is required. Consequently, deciphering the underlying molecular mechanisms may be conducive to identifying novel molecular biomarkers for diagnosis and surveillance.

Mass screening programs have facilitated the early detection of CRC, with the majority of tumors diagnosed at stages I or II. However, when CRC is identified based on clinical symptoms, tumors are often detected at more advanced stages (III or IV, according to the TNM classification). At these later stages, treatment becomes both costly and less effective, with poor patient outcomes due to tumor recurrence and/or resistance to therapy [3,4]. By focusing investigation on stage IV CRC, one would assume approaching host and/or environment factors involved in the failure of anti-tumor immune response [4,5].

Diet, the main environmental factor, has been shown to favor CRC development, primarily by affecting gut microbiota composition and causing dysbiosis, which subsequently impacts the host [6,7,8]. Diet-induced dysbiosis promotes mild chronic inflammation within the colonic mucosa, accelerating epithelial cell turnover and increasing cancer risk [9]. The proliferation of certain bacterial species, including *Fusobacterium* spp., *Bacteroides fragilis*, and *Escherichia coli*, is influenced by dietary factors, and their overabundance is associated with mucosal inflammation, genomic instability, and aberrant epigenetic modifications [8]. Furthermore, inflammatory responses triggered by specific bacterial taxa have been shown to alter immunosuppressive lipid metabolism, contributing to chemoresistance in CRC patients [10,11]. Efforts are also underway to elucidate the mechanisms underlying the failure of immunotherapy in the majority of colon and rectal cancers. Given the critical importance of predicting therapeutic response, whether in neoadjuvant or adjuvant course, we aimed to investigate microbial, metabolic, and proteomic profiles in a cohort of CRC patients prior to therapy.

In addition to microbial dysbiosis, metabolic alterations play a pivotal role in CRC pathogenesis. Quantitative and structural abnormalities in metabolite profiles have been associated with CRC development, influencing intestinal permeability and mucosal inflammation. However, only a limited number of relevant metabolites have been identified in stool samples, with even fewer detected in serum [12,13,14,15,16]. In this study, we conducted an untargeted metabolomic analysis across multiple biological fluids (stool, serum, and urine) from the same individuals. We analyzed both the metabolome of effluents and whole metagenome of stool from a cohort of CRC patients at different disease stages and compared these findings with a control group with normal colonoscopy. By investigating three effluents we identified key metabolic pathways involved in the complex interplay between microbiota, metabolism, and CRC pathogenesis. Among those, the tryptophan pathway was found to be the most significantly altered from early to most advanced stages of colon carcinogenesis.

## 2. Materials and Methods

### 2.1. Study Patient and Sample Collections

This study is a non-randomized, prospective, multicentric observational investigation designed for metabolomic and microbiota analyses, as well as ELISA evaluation. Patients and control participants were randomly selected from our biobank and assigned to one of five conditions, normal colonoscopy (Control) or colorectal cancer at any stage (I, II, III, or IV), provided they had not received antibiotics, chemotherapy, radiotherapy, or immunosuppressive therapy.

In brief, adult participants were recruited between 2005 and 2010 from endoscopy departments across various hospitals within the “Assistance Publique-Hôpitaux de Paris” network, where they had been referred for colonoscopy, as previously described [17,18]. The study protocol adhered to the ethical principles outlined in the World Medical Association Declaration of Helsinki and was approved by the Ethics Committee [“Comité Consultatif de Protection des Personnes dans la Recherche Médicale” (CPP Créteil-Henri Mondor)] for the “Île-de-France” region, registered under number BRM 04-02 on 2004 Feb by INSERM (assurance n° 887412003056 protocol 04-02, société Gerling 111 rue Longchamp 75016). All participants provided written informed consent, which was approved by the Ethics Committee.

To be eligible for inclusion in the study, individuals had to have no history of colorectal surgery, colorectal cancer, inflammatory or infectious intestinal conditions, and should not have required emergency care or antibiotic treatment in the month preceding enrollment. Any special diets or medications taken during this period were documented, as described elsewhere.

Blood, urine, and stool samples were collected prior to colonoscopy and stored at −80 °C in a biobank for subsequent analysis. Pathology reports and TNM classification were recorded in anonymized files, as described previously [17].

### 2.2. Metabolomics Analyses

All analytical-grade reference compounds used for metabolomic library were from Sigma (St Quentin Fallavier, France). The standard mixtures for the external calibration of the MS instrument (LTQ Velos ESI positive and negative calibration solutions, Pierce ThermoScientific, Courtaboeuf, France) were from Thermofisher Scientific (Courtaboeuf, France). Ammonium carbonate and ammonium hydroxide were from Sigma. Acetonitrile, methanol and water Optima^®^ LC/MS were from FisherScientific (Illkirch, France). Stock solutions of internal standards (1 mg/mL) were prepared in appropriate solvents. Then, mixtures of 8 compounds (aminoanthracene, amiloride, atropine, ethylmalonic acid, metformin, prednisone, ampicillin, and arachidonic-D8 acid) were prepared from each stock solution and diluted in methanol for HILIC analysis to achieve a final concentration of 20 µg/mL for each compound.

The HPLC chromatographic separations were performed on a ZICpHILIC® column (5 µm, 2.1, 150 mm length) at 15 °C (Merck, Darmstadt, Germany). Mobile phases were 10 mM of ammonium carbonate in water with addition of ammonium hydroxide to adjust basicity to pH 10.5 (phase B) and acetonitrile (phase A). Chromatographic elution started with an isocratic step of 2 min at 80% of acetonitrile, followed by a linear gradient from 80 to 40% of phase B from 2 to 12 min. The chromatographic system was then rinsed for 5 min at 0% B and the run ended with an equilibration step of 15 min at 80% phase A.

LC-MS/MS analyses were performed using an Ultimate 3000^®^ liquid chromatography system combined with a Q-Exactive Focus® mass spectrometer (Thermofisher Scientific, Courtaboeuf, France) fitted with an electrospray source operated in the positive and negative ion modes. The software interface was XCalibur® (2.1, Thermofisher, Courtaboeuf, France). The mass spectrometer was calibrated before each batch in both ESI polarities using the manufacturer’s recommendations. The Q-Exactive mass spectrometer operated with capillary voltage at −3kV in the negative ionization mode and 5 kV in the positive ionization mode. The capillary temperature was 280 °C. The sheath gas pressure and the auxiliary gas pressure were set at 60 and 10 arbitrary units with nitrogen gas, respectively. The mass resolution power of the analyzer was 50,000 m/Δ*m* and full width at half maximum (FWHM) at *m*/*z* 200. The detection was achieved from *m*/*z* 85 to 1000 in the two ionization modes.

For each matrix, quality control (QC) was obtained by pooling 20 µL of each sample to be studied. QC samples were extracted independently and injected several times throughout the series to assess the signal variations. A part of these pools was subsequently diluted 1/2, 1/4, and 1/8 using mobile phase mixture phase A/B, (40:60, *v*/*v*). Each diluted sample was injected three times in the beginning of the sequence to test the quality of signal.

On ice, 300 µL of each sample (QC, diluted QC, serum, urine, and fecal water patient samples) was spiked with 100 µL of internal standards solution at 20 µg/mL and submitted to protein precipitation using 1100 µL of methanol. The resulting samples were then mixed using a vortex mixer for 20 s, left on ice at 4 °C for 30 min to allow protein precipitation, then centrifuged for 20 min at 15,000 rpm. Supernatants of each extract were divided into three aliquots (positive and negative ionization modes and a stock sample) and dried under nitrogen. Dried extracts were stored at −80 °C prior to LC-HRMS analyses. Each dried extract was resuspended in 50 µL of phase A/B, 40:60 (*v*/*v*) for HILIC analysis.

### 2.3. Data Processing and Bioinformatic Analyses of Metabolites

All raw data were manually inspected using the Qualbrowser® module of Xcalibur®. Raw files were first converted to mzXML format using the MSconvert® software (Apache-2.0, Knoxville, TN, USA). Automatic peak detection and integration were performed using the XCMS software package on the W4M® platform (INRAE, Paris, France) [19], which returned a data matrix containing *m*/*z* and retention time values of features together with their concentrations expressed in arbitrary units (i.e., areas of chromatographic peaks). XCMS features were thereafter filtered according to the following criteria: (i) the correlation between dilution factors of QC samples and areas of chromatographic peaks (filtered variables should exhibit coefficients of correlation above 0.7 in order to account for metabolites occurring at low concentrations and which are not detected anymore in the most diluted samples), (ii) repeatability (the coefficient of variations obtained for chromatographic peak areas of QC samples should be below 30%), and (iii) ratio of chromatographic peak area of biological to blank samples above a value of 3. Chromatographic peak areas of each variable present in the XCMS peak lists were normalized using the LOESS algorithm to remove analytical drift induced by clogging of the ESI source observed during analytical runs.

Features were annotated thanks to our internal spectral database according to accurately measured masses and chromatographic retention times obtained from ~1000 pure authentic standards [20] and to public databases (KEGG (Kyoto Encyclopedia of Genes and Genomes, Japan), HMDB (The Human DataBase, Canada), and Metlin (Metabolite and Chemical Entity Database, USA)). Confirmation of metabolite annotation was then accomplished by running additional LC-MS/MS experiments. Resulting tandem mass spectra were matched by those included in our in-house spectral database. To be identified, metabolites had to match at least 2 orthogonal criteria (accurately measured mass, isotopic pattern, MS/MS spectrum, and retention time) to those of an authentic chemical standard analyzed under the same analytical conditions.

After log2 transformation, intensity values were normalized by median centering within conditions (normalized function of the R package DAPAR) [21]. Missing values were imputed using the imp. norm function of the R package norm (norm: Analysis of multivariate normal datasets with missing values, 2013 R package (version 1.0–9.5)). A Limma t-test was applied to determine metabolites with a significant difference in abundance while imposing a minimal fold change of 2 between the conditions to conclude that they are differentially abundant [22,23]. An adaptive Benjamini–Hochberg procedure was applied on the resulting p-values using the function adjust.p of R package cp4p and the robust method described in Pounds et al. [24] to estimate the proportion of true null hypotheses among the set of statistical tests. The metabolites with an adjusted *p*-value inferior to a False Discovery Rate (FDR) of 0.01 or less were considered as differentially significant. The enrichment analysis has been performed using Metaboanalyst®5.0 (Ste Anne de Bellevue, QC, Canada) [25].

### 2.4. Microbiota Analysis

Whole-genome shotgun sequencing of fecal samples collected in France was performed using the Illumina HiSeq 2000/2500 (Illumina, San Diego, CA, USA) as described in detail elsewhere [26]. Paired-end sequencing with a read length of 100 bp was conducted at the Genomics Core Facility, European Molecular Biology Laboratory, Heidelberg. Taxonomic profiling was carried out using the DIAMOND + MEGAN pipeline with the NCBI-nr protein database and default setting (Diamond v-0.8.36, [27]; MEGAN6 v6.7.11, [28], (Tubingen, Germany)).

A subset of 73 out of 156 samples from the French cohort, for which both metabolomic profiles and species-level taxonomic abundance data were available, was used for microbial–metabolite network reconstruction. Species abundances were normalized as relative percentage, while metabolite abundances were log_10_-transformed. The following filters were applied prior to network analysis: (1) only species with a relative abundance greater than 0.01% (n = 49 out of 1022) were retained and (2) species or metabolites with a prevalence below 40% within the group were excluded.

Species–metabolite co-occurrence networks were constructed using both Pearson and Spearman correlation coefficients. Statistical significance of correlations was assessed via 1000 permutations. Correlations were considered significant if the associated *p*-value was <0.05 and the absolute Pearson correlation coefficient exceeded 0.3. The resulting species–metabolite interaction networks were visualized using the Cytoscape software (Cytoscape® v3.10.2 (New York, NY, USA) [29]). In the graphical representation, nodes correspond to microbial species or metabolite, with node size proportional to the fold change (FC; Cancer vs. Control). Edge represents correlations, with green indicating positive correlations (co-occurrence) and blue indicating negative correlations (co-exclusion).

### 2.5. Quantification of Inflammatory Biomarkers and Statistical Analysis

Interleukin (IL)-8, IL-10, IL-18, and TNF-alpha were measured using an enzyme-linked immunosorbent assay (Quantikine R&D Systems, Oxford, UK). Haptoglobin, α1glycoprotein acid, albumin, and S100A8/A9 (serum calprotectin) were quantified using a turbidimetric assay (Cobas 501, Roche, Meylan, France), high-sensitivity C-reactive protein (Hs-CRP) by immunonephelometry (IMMAGE, Beckman-Coulter, Villepinte, France), and IL-6 was quantified using a chemiluminescent assay (Cobas 8000, Roche). External quality controls were regularly performed, and analytical methods were validated according to international quality recommendations (ISO 15189, Cofrac, Paris, France). All quantitative results were expressed as median and interquartile and statistical analysis performed using Mann–Whitney test with differences considered as significant for *p* < 0.05.

## 3. Results

### 3.1. Characteristics of Individuals

A total of 53 patients with colorectal cancer (CRC) and 20 control individuals were included in the present study (Table 1).

**Figure 1:**
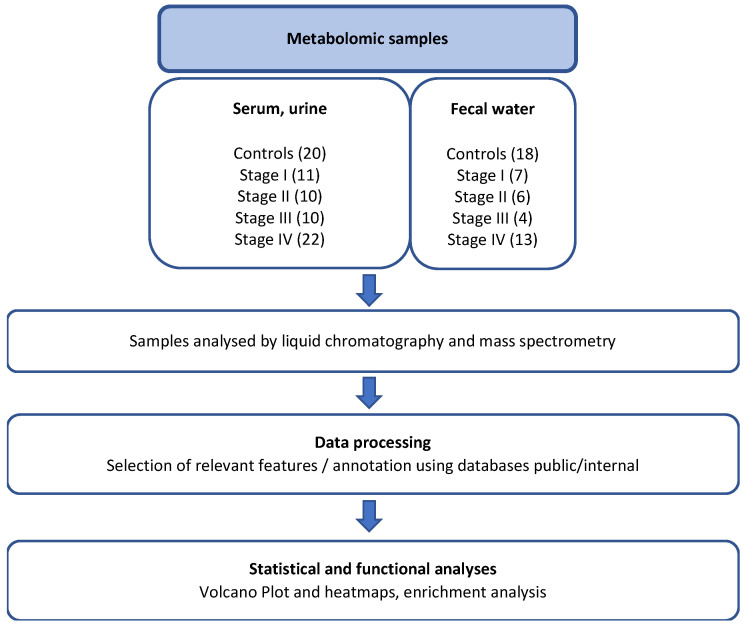
Design of the study and general overview.

The patient cohort consisted predominantly of women (n = 24), with a median age of 67 years (range: 44–87) and a median body mass index (BMI) of 25.3 kg/m^2^ (range: 15–40). Based on the international TNM classification, and assuming that patients with more advanced tumors exhibit a higher degree of immune evasion, 22 stage IV patients (CRC with synchronous metastasis; 41% of current CRC cases) were compared to subgroups of stage I (n = 11, 21%), stage II (n = 10, 19%), and stage III (n = 10, 19%) CRC patients, as well as to controls with normal colonoscopy findings (n = 20). Metabolite abundances in various biological effluents from each patient were determined, and mean values for each subgroup were compared to those of control individuals through unsupervised analyses (Figure 1).

CRC patients and controls were matched for BMI, although CRC patients were significantly older (mean age: 66.2 ± 12 vs. 57.5 ± 20 years; Table 1) and women outnumbered men.

### 3.2. Metabolomic Profiling in CRC Patients

Following metabolite trimming and annotation, 166 metabolites were found to be significantly altered in CRC patients compared to controls, as determined by unsupervised analysis. Specifically, 59 metabolites in serum, 72 in urine, and 35 in fecal water were differentially expressed (Tables S1–S4, stratified by CRC stage). Notably, 11 isomeric metabolites could not be precisely identified due to unresolved chromatographic peaks in the liquid chromatography–high-resolution mass spectrometry analysis (Table S5).

Metabolites involved in amino acid, fatty acid, and lipid metabolic pathways exhibited differential expressions between CRC patients and controls. Based on fold-change values, these alterations varied by CRC stage. In early-stage CRC (stages I–II), ten urinary metabolites and four fecal water metabolites were significantly different (Table 2).

**Figure 2:**
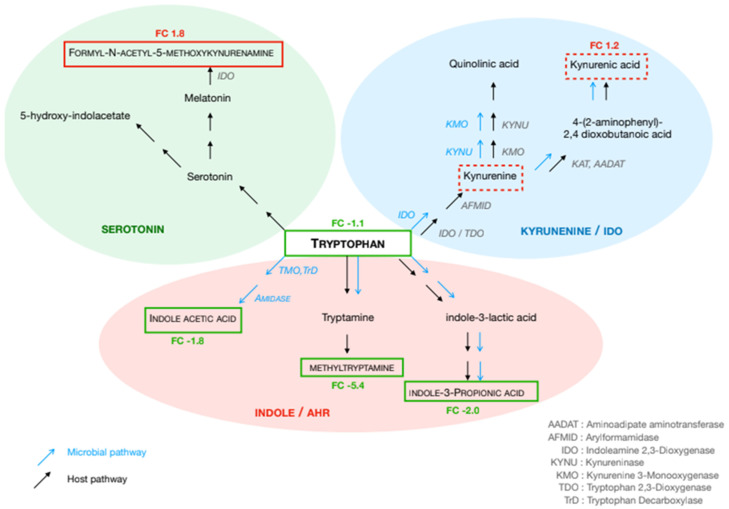
Pathways of tryptophan metabolism through the serotonin, kynurenine and indole/AhR pathways. Metabolites written in caps are those significantly different in each effluent of cancer patients. Metabolites framed in red were significantly higher in stage IV cancer patients than in controls (Formyl-N-Acetyl-5-methoxykynurenine in serum and Indole-3-lactic acid in urine), while those in green were significantly lower (tryptophan and indole-3-propionic acid in serum and methyltryptamine in fecal water).

In stage III patients, 10 metabolites in both serum and urine were altered, all of which were linked to tryptophan metabolism. In stage IV patients, hierarchical clustering revealed distinct metabolite expression patterns across serum, urine, and fecal water samples. Fifteen top-ranked metabolites were involved in amino acid metabolism (methylhistidine, glutamate, arginine, tryptophan, and ornithine), N-acetylspermidine, lipidic compounds, pentose, and peptides. However, metabolite levels in serum samples exhibited some degree of overlap between CRC patients and controls (Figure 2).

Furthermore, in stage IV CRC patients, serum concentrations of linoleic acid (fold change [FC]: −1.07), deoxycholic acid (FC: −1.53), and cholic acid (FC: −2.44) were significantly lower, while arachidonic acid levels were elevated (FC: 1.49). Overall, lipid catabolites, including bile acids, steroid conjugates, and fatty acyls, were found at higher levels in stage IV CRC patients across serum, urine, and fecal water samples compared to controls (Table S4). Specifically, bile acid metabolites derived from taurine and glycine were elevated in the urine of stage IV CRC patients. Additionally, metabolic alterations were observed in the pentose phosphate and glycerol phosphate pathways in both serum and fecal water, as well as changes in phosphatidylethanolamine and phosphatidylcholine biosynthesis in serum. Metabolites related to vitamin B6 and sphingolipids were differentially expressed in both serum and urine samples.

### 3.3. In-Depth Analysis of Amino Acid Metabolism in Stage IV CRC Patients

Significant modifications regarding amino acids involved in the immune responses were observed. Notably, significant shifts in the tryptophan (Trp) pathway were observed in stage IV CRC patients as compared to controls (Figure 2). Trp levels were reduced in both serum (FC: −1.12) and urine (FC: −1.6), whereas kynurenate levels were higher in these effluents (FC: 1.2 and FC: 1.4, respectively). Indole acetic acid and indole-propionic acid concentrations were lower in serum (FC: −1.8 and FC: −2.0, respectively), while methyltryptamine was decreased in fecal water (FC: −5.4). Regarding the serotonin pathway, formyl-acetyl-5-methoxy-kynurenine increased in serum (FC: 1.3), while hydroxy-indoleacetate showed a decreasing trend in urine (FC: −1.1). In the histidine pathway, histidine (FC: −2.12 in serum; FC: −1.2 in urine), methyl-histidine (FC: −1.81 in serum; FC: −1.02 in urine), imidazole-4-acetate (FC: −2.05 in serum; FC: −1.92 in fecal water), and histidine methyl ester (FC: −2.27 in fecal water) were decreased, whereas carnosine was elevated in urine (FC: +1.5).

In the glutamate pathway, glutamate (FC: +1.16), N-acetylglutamate (FC: +1.51), and β-citryl-glutamate (FC: +1.51) were elevated in serum, whereas N-acetyl-ornithine was decreased in both serum (FC: −1.03) and fecal water (FC: −1.53) and N-acetylglutamate significantly decreased (FC:−1.58) in urine. Additionally, 2-amino-α-dioxobenzenebutanoic acid levels were lower in fecal water (FC: −2.9).

Across all CRC patients, N-acetylalanine (FC: +1.32) and N8-acetylspermidine (FC: +1.29) were increased in urine and serum, respectively. Guanidinoacetate levels were reduced in both serum (FC: −1.0) and urine (FC: −1.43).

### 3.4. Biomarkers of Inflammation

**Figure 3:**
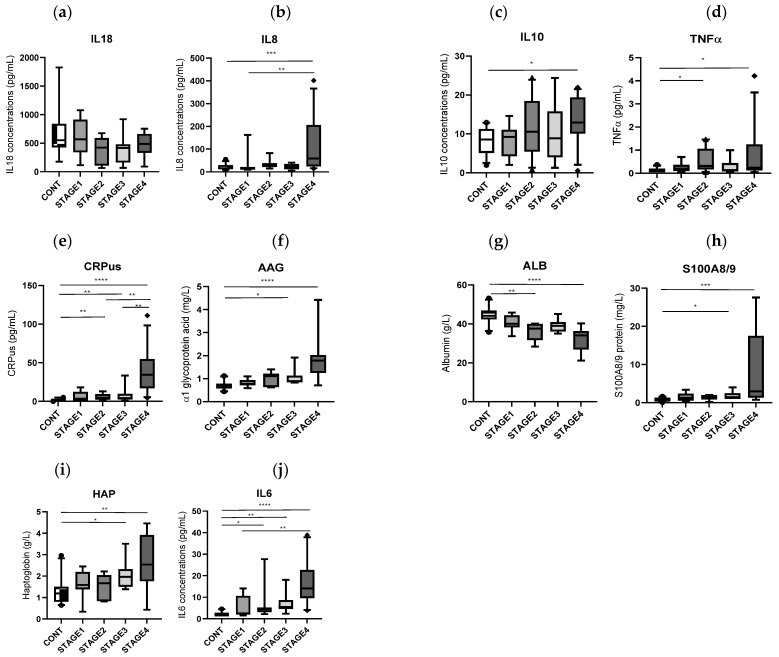
Quantitative analysis of inflammatory cytokines and proteins in controls (n = 12), in stage I cancer (n = 8), in stage II cancer (n = 10), in stage III cancer (n = 9), and in stage IV CRC patients (n = 12) [Median, 10–90 percentile]. Comparison: 0. * *p* < 0.5, ** *p* < 0.01, *** *p* < 0.001, **** *p* < 0.0001. (**a**): Interleukin-18, (**b**): Interleukin-8, (**c**): Interleukine-10, (**d**): Tumor Necrosis Factor-α, (**e**): C protein ulrasensible, (**f**): alpha-acid glycoprotein, (**g**): albumin, (**h**): S100A8/9, calprotectin, (**i**): haptoglobin, (**j**): Interleukin-6.

Inflammatory markers in the effluents exhibited significant alterations in CRC patients as compared to controls (Figure 3), particularly in stage IV cases. TNF-α levels were slightly elevated in stage IV (median: 0.23 vs. 0.14 pg/mL, *p* = 0.04) and stage II (median: 0.3 vs. 0.14 pg/mL, *p* = 0.013) CRC patients. High-sensitivity CRP (hs-CRP) was significantly increased in stage IV (34.3 vs. 1.1 µg/mL, *p* < 0.0001) and stage III (4.1 vs. 1.1 µg/mL, *p* = 0.013).

As compared to controls, interleukin-6 (IL-6) levels were elevated in stage IV (median: 14.1 pg/mL, *p* < 0.0001), stage II (median: 4.5 vs. 1.6 pg/mL, *p* = 0.033), and stage III (median: 5.5 pg/mL, *p* = 0.03), and IL-8 (median: 80.5 vs. 16 pg/mL, *p* = 0.0005) and IL-10 (median: 13.7 vs. 8.6 pg/mL, *p* = 0.021) were significantly elevated in stage IV CRC patients. Several inflammatory proteins including α1-acid glycoprotein (AAG), haptoglobin, and S100A8/A9 (*p* < 0.001 for all), as well as albumin levels were significantly lower (34.1 vs. 44.1 g/L, *p* < 0.001) in stage IV CRC patients than in controls.

### 3.5. Microbial Metabolites and Stool Microbiome Analysis

Several microbial-derived metabolites exhibited significant alterations in serum samples from patients with stage IV colorectal cancer (CRC) compared to healthy controls. Specifically, significant decreases were observed in dihydroxybenzoate (fold change [FC]: −1.9), 2,5-furandicarboxylic acid (FC: −2.38), hydroxyphenyllactate (FC: −1.55), 5-methyl-2-furancarboxaldehyde (FC: −2.14), and methylxanthine (FC: −2.76).

When analyzing all samples collectively, the distribution of enterotypes, as a descriptor of global community structure was found to be different between CRC patients and controls [26]. Taxonomic profiling of the fecal microbiota revealed significant associations between several bacterial taxa and CRC status, with microbial dysbiosis shown to correlate with tumor staging based on the TNM classification.

**Figure 4:**
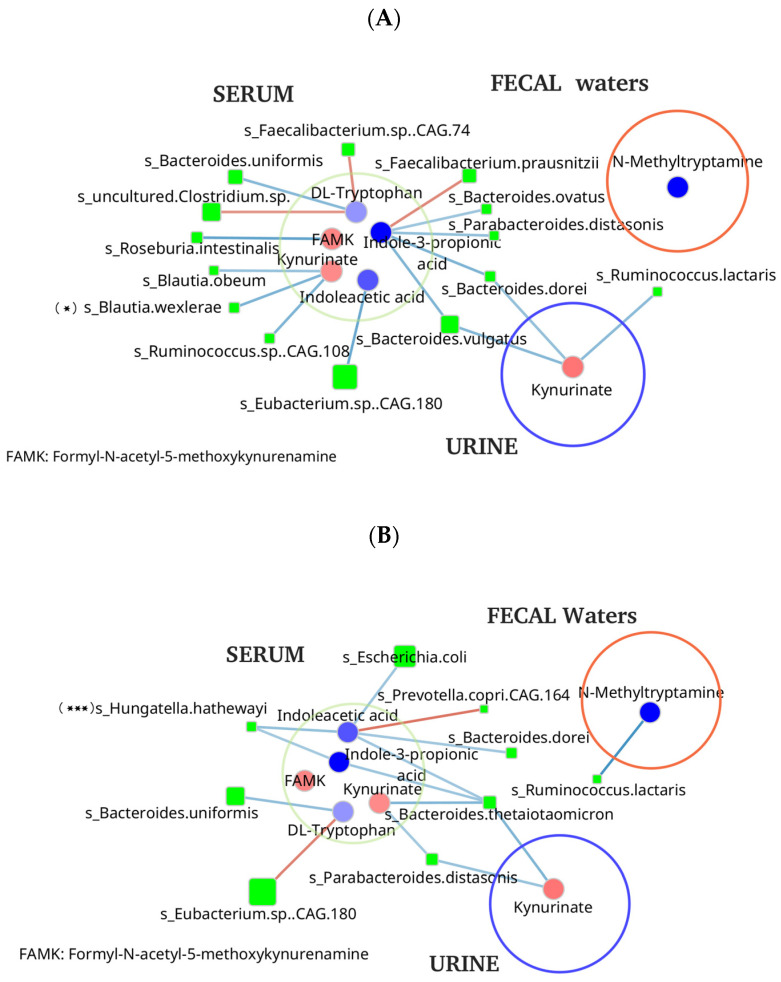
Correlations between differential metabolites of tryptophan and relative abundances of bacterial species, (**A**) for control group and (**B**) for stage-IV CRC group, in serum, urine, and fecal water. Each node represents a bacterial species, with node size proportional to its average relative abundance across the dataset. Significant associations between bacterial species and metabolites were determined using Pearson correlation analysis. Positive correlations (Pearson ’s ρ > 0.3, *p* < 0.05) are indicated by red edges, signifying that higher bacterial abundance is significantly associated with higher metabolite abundance. Negative correlations (Pearson ’s ρ < −0.3, *p* < 0.05) are indicated by blue edges, reflecting an inverse relationship where lower bacterial abundance is significantly associated with higher metabolite abundance. (*) s_Blautia was significantly decreased in stage IV cancer group; (***) s_Hungatella was significantly increased in stage IV cancer group.

A co-occurrence network, constructed using the relative abundances of differentially abundant bacterial species and metabolites, revealed distinct differences between control individuals and stage IV CRC patients across all three effluents currently analyzed (Figure 4).

Dysbiosis appeared to progress with disease stage, with a notable enrichment of *Escherichia/Shigella* species in stage III–IV CRC and a corresponding depletion of *Bifidobacterium* and *Ruminococcus* spp. in both early and advanced disease stages. *Blautia* spp. were significantly reduced in stage IV patients, while *Hungatella* spp. were significantly increased compared to controls.

Network analysis also uncovered differential microbial-metabolite associations between stage IV CRC patients and controls, particularly among metabolites associated with cancer-related alterations (Figure 4). No significant associations were identified with metabolites from other metabolic pathways.

In the control group, certain symbiotic bacteria, such as *Faecalibacterium* spp., *Ruminococcus*, and *Bacteroides* spp., showed strong associations with metabolites from tryptophan metabolic pathway, including tryptophan itself, kynurenate, indole-3-propionic acid, and indole acetic acid (Table S7) [12,18,26]. In contrast, in the stool of stage IV CRC patients, virulent genera such as *Escherichia*, *Hungatella*, and *Bacteroides* were associated with tryptophan-derived metabolites in the serum of stage IV CRC patients, suggesting a shift in microbial contributions to host tryptophan metabolism, whereas several symbiotic bacteria, including *Faecalibacterium* and *Eubacterium*, were predominant in controls and no similar significant associations with tryptophane metabolites were observed in controls (Table S7).

## 4. Discussion

Chronic low-grade inflammation within the normal mucosa serves as a precursor to carcinogenesis, persisting throughout disease progression from early-stage colorectal cancer (CRC) to metastasis. This prolonged inflammatory state, whether driven by chronic host–microbiota interactions or dietary factors such as protein-rich consumption, contributes to age-related metabolic alterations. As a result, metabolites, the end products of these processes, can serve as critical biomarkers of microbe–host interactions, potentially identifying individuals at higher risk for CRC development [6,30,31].

In this study, we identified a panel of metabolites in serum and urine that were linked to key inflammatory markers, including TNF-α, CRP, IL-6, IFN-γ, and IL-12, distinguishing CRC patients from non-cancerous controls. The strength of our approach lies in the simultaneous analysis of three biological matrices—serum, urine, and fecal water—across different CRC stages, allowing for comprehensive metabolic profiling. In agreement with previous studies, we observed that the number of differential metabolites increased with CRC severity, as assessed by TNM staging [14,32,33]. Notably, our findings highlight significant catabolism of amino acids, particularly tryptophan, histidine, and glutamate, which play pivotal roles in regulating the immune response to tumors.

Tryptophan metabolism has emerged as a key pathway associated with poor outcomes, particularly in stage IV CRC patients. As an essential amino acid, tryptophan is involved in protein synthesis and can be metabolized into serotonin, indole derivatives, and kynurenine [33,34]. Our results indicate that tryptophan catabolism is dysregulated from stage II to stage IV, alongside elevated levels of proinflammatory cytokines (IL-6, TNF-α, and CRP) and an overabundance of virulent bacterial species such as *Escherichia coli* in stool samples [35]. This suggests a critical role for these virulent bacteria in triggering and sustaining inflammatory pathways that drive tumor progression [35,36]. Given that tryptophan metabolism is tightly linked to immune regulation, we performed an in-depth analysis of its metabolites in relation to stool bacterial composition [37,38,39].

The elevated kynurenine levels observed in the serum and urine of stage IV CRC patients indicate enhanced tryptophan degradation, which is known to suppress T-cell proliferation and promote regulatory T-cell expansion and immune tolerance [40]. Moreover, kynurenine activates the aryl hydrocarbon receptor (AhR), further contributing to immune suppression. The enzyme indoleamine 2,3-dioxygenase 1 (IDO1), which mediates this pathway, is highly expressed in tumor tissues and promotes tumor growth by facilitating immune escape [41]. Our findings align with previous studies demonstrating that IDO1 expression correlates with reduced infiltration of CD3+, CD8+ T cells, and CD57+ natural killer cells within tumor tissues [42]. Additionally, indole derivatives such as indole acetic acid and indole propionic acid, both AhR ligands, may compete for receptor binding, further influencing immune modulation [43]. This underscores the critical role of gut microbiota in regulating AhR expression and function in tumor and inflammatory cells. Recently, it has been described that AhR activation promotes intestinal barrier repair and spermidine rescues intestinal barrier defects in mice with colitis via AhR-Nfr2 and AhR-Stat3 pathways; this could explain the increasing of acetylspermidine [44].

Our results also align with findings from healthy individuals where kynurenine levels positively correlate with type 1 immunity markers (TNF-α, CRP, and IL-10) and symbiotic gut bacteria, including *Lachnospiraceae*, *Dorea*, and *Ruminococcus*. In contrast, our microbial analysis in the stool of CRC patients revealed a significant reduction in symbiotic bacteria such as *Ruminococcus* and *Bifidobacterium*, particularly in early-stage CRC, coupled with an increase in pro-inflammatory genera such as *Escherichia* and *Desulfovibrio*. These findings suggest that an imbalance between symbiotic and virulent bacteria may serve as a pivotal marker of tryptophan-metabolism-related immune dysfunction in CRC [39,42].

Beyond tryptophan metabolism, other amino acids such as glutamine and arginine are also modulated by gut microbiota. Glutamate, a key product of glutaminase activity, is elevated in cancer tissues, plants, and various bacterial species [45]. Similarly, methylhistidines, branched-chain amino acids (BCAAs), and aromatic amino acids—known risk factors for cancer in young individuals—are of particular interest in understanding resistance to chemotherapy. Chemoresistance, along with immune evasion within tumors, increases the risk of metastasis and contributes to poor clinical outcomes [46]. These observations support the need for dietary intervention trials aimed at preventing CRC recurrence and for immune-targeted therapies that reshape the gut microbiota.

Furthermore, bacteria adhering to tumor tissues exhibit peptidase enzymatic activity, leading to polyamine accumulation in tumor cells. Polyamines, which are essential for cell division, disrupt phospholipid metabolism—a process highly conserved in bacterial species. Notably, certain *Bacteroidetes* species produce sphingolipids, which play a crucial role in immune system maturation. However, their precise physiological and metabolic roles in host disease remain poorly understood [47]. Our study found significant alterations in sphingolipid metabolites in urine, particularly in stage IV CRC patients. Concurrently, urinary bile acid metabolites were highly elevated, whereas their detection in stool samples proved challenging due to hydration-related variability. This highlights the complexity of distinguishing between eukaryotic and microbial metabolite origins.

Despite the comprehensive nature of our multi-matrix approach integrating both prokaryotic and eukaryotic metabolomics, certain limitations must be acknowledged. As a pilot study, our cohort size was relatively small, particularly for subgroup analyses. Additionally, we did not perform specific analytical pretreatment for lipid and sugar metabolites, which may have led to underestimation of these pathways. However, our findings demonstrate that tryptophan catabolites are detectable in urine at early CRC stages, supporting the potential use of urinary biomarkers for CRC mass screening programs. In contrast, at advanced tumor stages (III and IV), metabolites were more readily detectable in blood, suggesting that serum metabolite profiling may be essential for monitoring therapeutic responses in patients undergoing chemotherapy [48].

## 5. Conclusions

In conclusion, we describe a disease-associated network connecting *E. coli* with microbial-associated tryptophan metabolism as the main pathway altered in most advanced CRC patients with poor outcomes. Tryptophan/kynurenine metabolites analysis can be used as a marker in urine or serum to screen for colon cancer dysbiosis-related immune response under therapy.

## Figures and Tables

**Table 1:** Characteristics of CRC patients and healthy controls (median [quartiles]).

Characteristic	Control	CRC Patients	*p* Value
Individuals, n	20	53	
Gender (n, %)			
Female	13 (65%)	24 (45%)	
Male	7 (35%)	29 (55%)	
Age (yr)	59	67	0.005
(extremes)	(35–77)	(44–87)	
BMI (kg)	25.5 (20–34)	25.3 (15–40)	0.618
TNM staging (n, %)		53	
I	-	11 (21%)	
II	-	10 (19%)	
III	-	10 (19%)	
IV	-	22 (41%)	
Tumor location (n, %)		Number (%)	
*Right colon*	-	21 (40%)	
*Left colon*	-	21 (40%)	
*Rectum*	-	11 (20%)	
Molecular markers in tumor tissues (n, %)		2 (4%)	
*MSI*	-	45 (84%)	
*WIF-1 Gene methylated*	-	22 (42%)	
*KRAS mutated*	-	0	
Braf mutated			

**Table 2:** Top 10 upregulated and downregulated metabolites in effluents of CRC patients versus controls.

Metabolites	Log2FC Cancer II/Controls	Log2FC Cancer III/Controls	Log2FC Cancer IV/Controls
**Serum**			
Cotinine	2.2873		
Ser-Gly-Thr/Ala-ser-ser/Thr-ser-Gly	2.0674	2.2671	3.2037
Acetaminophen			3.2661
Aspartylphenylalanine			1.5630
N-Acetyl-L-glutamic acid			1.5179
Eicosanoids			1.4908
beta-citryl-L-glutamate			1.5133
N8-Acetylspermidine			1.2949
4-Hydroxy-2-quinolinecarboxylic acid (Kynurinate)		2.9540	1.2238
Dglutamic acid			1.1606
Cholic acid	−2.6064		−2.4377
2,5-Furandicarboxylic acid	−2.2914		−2.3831
gamma-glutamyl-Se-methylselenocysteine	−2.2428		−2.6907
(9Z,11E)-(13S)-13-Hydroxyoctadeca-9,11-dienoic acid	−2.1660		
13 carboxygamma tocopherol	−2.1075	−2.1619	−2.8619
2-OH-3(2,3,4-trimethoxyphenyl)propanoic acid			−3.1326
7-Methylxanthine/1-Methylxanthine/3-Methylxanthine			−2.7637
Ethyl 2-(methyldithio)propionate			−2.4824
Guanidinosuccinic acid			−2.2632
5-methyl-2furancarboxyaldehyde (5-methyl-2-furfural)			−2.1402
**Urine**			
Galactonic acid/Gluconic acid	2.6300		
pcresol sulfate		2.1906	1.2992
4-Hydroxy-2-quinolinecarboxylic acid (Kynurinate)		2.0819	1.4458
Sphingosine 1-phosphate			2.7050
Taurochenodeoxycholate 3/7-sulfate			2.5328
Sulfoglycolithocholate/sulfolithocholylglycine			1.9500
Taurolithocholic acid-3-sulfate			1.9476
N6-(L-1,3-Dicarboxypropyl)-L-lysine (Saccharopine)			1.9295
Glucosylgalactosyl hydroxylysine			1.8288
5-Methoxyindoleacetate/Indolelactate			1.7167
L-Tartaric acid	−4.5342		
{[3-(2-hydroxy-4-methoxyphenyl)prop-2-en-1-yl]oxy}sulfonic acid ou [1-(4-methoxyphenyl)-3-oxopropan-2-yl]oxy}sulfonic acid	−3.4328	−2.7478	−2.9375
N6-Methyladenine	−2.8901		−1.6964
N-acetyl-L-arginine	−2.7452		
D-(+)-Neopterin	−2.6057	−2.1259	−2.5797
N-Acetyl-L-glutamic acid	−2.5539		−1.5816
Deoxyinosine	−2.5367		−1.6937
L-Histidine	−2.5125		
4-Phenoxybutyric acid/butanoic acid	−2.4893		
4-Hydroxycoumarin/4-hydroxy-2H-chromen-2-one			−3.1658
**Fecal water**			
Acetylcysteine	−6.0085		−5.4812
Hypoxanthine	−3.8516		
13 carboxygamma tocopherol	−3.5300		−4.8583
Citric acid	−2.3928		
N-Methyltryptamine			−5.3552
N-(3-methylbutyl)-acetamide			−4.6979
2,3-dihydrobenzofuran			−3.3037
DL-p-Hydroxyphenyllactic acid/Homovanillic acid			−3.0083
Tyrosyl-Isoleucine			−3.0111
4(2-aminophenyl)-2,4 dioxobutanoic acid			−2.8709
Valsartan			8.8180
Aspartylglutamate			5.0664
Acetyltaurine			3.6545
N-Acetylneuraminic acid			3.4861

## Data Availability

The original contributions presented in this study are included in the article/supplementary material. Further inquiries can be directed to the corresponding author.

## Supplementary Materials

The following supporting information can be downloaded at: https://www.mdpi.com/article/10.3390/biomedicines14010026/s1, Table S1. (A): The annotated metabolites identified in urine of stage I CRC patients and controls; (B): the annotated metabolites identified in fecal water of stage I CRC patients and controls. Table S2. (A): The annotated metabolites identified in serum of stage II CRC patients and controls; (B): the annotated metabolites identified in urine of stage II CRC patients and controls; C: the annotated metabolites identified in fecal water of stage II CRC patients and controls. Table S3. (A): The annotated metabolites identified in serum of stage III CRC patients and controls; (B): the annotated metabolites identified in urine of stage III CRC patients and controls. Table S4. (A): The annotated metabolites identified in serum of stage IV CRC patients and controls; (B): the annotated metabolites identified in urine of stage IV CRC patients and controls; (C): the annotated metabolites identified in fecal water of stage IV CRC patients and controls. Table S5. List of metabolite peaks which were identified in the present study and could correspond to more than one annotated metabolite. Table S6. List of metabolite–microbial correlation. Table S7. Significant correlation between microbial species and tryptophan metabolites.

## Abbreviations

The following abbreviations are used in this manuscript:

CRC	Colorectal cancer
IL	Interleukin
Trp	Tryptophan
Kyn	Kynurenin
CRP	C-Reactive Protein
TNF	Tumor Nuclear Factor
IDO1	Indoleamine 2,3 dioxygenase 1
AhR	Aryl hydrocarbon Receptor
